# Orai1 promotes tumor progression by enhancing cancer stemness *via* NFAT signaling in oral/oropharyngeal squamous cell carcinoma

**DOI:** 10.18632/oncotarget.9755

**Published:** 2016-06-01

**Authors:** Sung Hee Lee, Nicole Kristina Rigas, Chang-Ryul Lee, April Bang, Sonal Srikanth, Yousang Gwack, Mo K. Kang, Reuben H. Kim, No-Hee Park, Ki-Hyuk Shin

**Affiliations:** ^1^ The Shapiro Family Laboratory of Viral Oncology and Aging Research, UCLA School of Dentistry, Los Angeles, CA, USA; ^2^ Department of Physiology, David Geffen School of Medicine at UCLA, Los Angeles, CA, USA; ^3^ UCLA Jonsson Comprehensive Cancer Center, Los Angeles, CA, USA; ^4^ Department of Medicine, David Geffen School of Medicine at UCLA, Los Angeles, CA, USA

**Keywords:** Orai1, OSCC, cancer stem cells, NFAT, SOCE

## Abstract

Emerging evidence indicates that Orai1, a key calcium channel for store-operated Ca^2+^ entry, is associated with human cancer. However, the underlying mechanism by which Orai1 regulates cancer progression remains unknown. Here we report that intracellular level of Orai1 is increased in a stepwise manner during oral/oropharyngeal carcinogenesis and highly expressed in cancer stem-like cell (CSC)-enriched populations of human oral/oropharyngeal squamous cell carcinoma (OSCC). Ectopic Orai1 expression converted non-tumorigenic immortalized oral epithelial cells to malignant cells that showed CSC properties, *e.g.,* self-renewal capacity, increased ALDH1^HIGH^ cell population, increased key stemness transcription factors, and enhanced mobility. Conversely, inhibition of Orai1 suppressed tumorigenicity and CSC phenotype of OSCC, indicating that Orai1 could be an important element for tumorigenicity and stemness of OSCC. Mechanistically, Orai1 activates its major downstream effector molecule, NFATc3. Knockdown of NFATc3 in the Orai1-overexpressing oral epithelial cells abrogates the effect of Orai1 on CSC phenotype. Moreover, antagonist of NFAT signaling also decreases CSC phenotype, implying the functional importance of Orai1/NFAT axis in OSCC CSC regulation. Our study identifies Orai1 as a novel molecular determinant for OSCC progression by enhancing cancer stemness, suggesting that inhibition of Orai1 signaling may offer an effective therapeutic modality against OSCC.

## INTRODUCTION

OSCC is the sixth most common cancer and an important public health concern worldwide [1]. Risk factors for OSCC development in the elderly are typically associated with a life-long history of cigarette smoking and heavy alcohol consumption [2, 3]. OSCC is often preceded by clinically well-defined lesions, such as leukoplakia that is histologically classified as dysplastic or non-dysplastic leukoplakia. Dysplastic leukoplakia is defined as oral premalignant lesion and associated with a likely progression to cancer; however, it is not an accurate predictor of cancer risk [4, 5]. Early stage tumors can usually be managed through surgery and radiotherapy. However, successful treatment is inversely proportional to the extent of the disease at the time of treatment. A combination of chemotherapy and radiation therapy, although effective in treating 97% of early stage tumors, was only 33% effective in treating advanced tumors [6]. In fact, the clinical stage at which the diagnosis is made is the most important prognostic indicator of oral cancer. Clearly, identifying the biomarker that allows detection of early stage cellular aberration leading to OSCC tumorigenesis is critically important for reducing of cancer-associated morbidity.

Recent studies have unveiled and validated the pathophysiologic role of CSCs (alternatively cancer initiating cells) in long-term sustenance of cancers [7]. CSCs are small subpopulations of tumor cells with self-renewal capacity and share many molecular similarities to embryonic and normal adult stem cells. CSCs have been isolated from various primary tumors and established cancer cell lines, including OSCC [8, 9]. They are also potentially responsible for drug resistance, metastasis, and recurrence of cancers. Therefore, CSCs drive the perpetuity of the disease while producing cellular heterogeneity of cancer tissues, making them strategically plausible targets for cancer therapies. The phenotypes of CSC have been reported to be maintained by several endogenous signaling pathways, such as Notch, Hedgehog, and Wnt [10, 11], which are frequently activated in human cancers [11–13]. In addition, increasing numbers of publications reported that CSCs could be epigenetically regulated by microRNAs [14, 15]. Therefore, advancing our understanding of the molecular properties and signaling pathways unique to OSCC CSCs is crucial for developing a new generation of targeted and effective therapies for oral/oropharyngeal cancer.

Calcium (Ca^2+^) plays indispensable roles in cell signaling pathways that involved in maintaining and regulating in normal biological processes [16–18]. Ca^2+^ homeostasis is disrupted during carcinogenesis, thereby leading to deregulated cell proliferation, migration and suppression of apoptosis [19–22]. In most of non-excitable cells, Ca^2+^ influx is tightly regulated by the store-operated Ca^2+^ entry (SOCE) pathway and mediated through store-operated Ca^2+^ release-activated Ca^2+^ (CRAC) channels [23]. Orai1 is an essential pore-subunit of the CRAC channels [24–26]. Upon stimulation, the cells release Ca^2+^ from the endoplasmic reticulum (ER) followed by extracellular Ca^2+^ influx through SOCE. SOCE not only refills the depleted ER Ca^2+^ stores but also provides a direct Ca^2+^ signal to activate downstream responses including the nuclear factor of activated T cells (NFAT) signaling pathway [27, 28]. Orai1 has been extensively studied in immunology as NFAT is the transcription factor that is necessary for activation, differentiation, and effector functions of T cells [29].

Currently, emerging evidence has indicated the efficacy of targeting Orai1to inhibit cancer growth and metastasis [30–34]. Orai1 was overexpressed in various human cancers, including lung, esophagus, brain, and kidney [33–35]. Clinically, Orai1 overexpression strongly linked to poor overall and recurrence-free survival in human esophageal SCC [36, 37] and non-small cell lung cancer [35]. Orai1 promoted cancer cell migration/invasion [30], drug resistance [38] and angiogenesis [39, 40], three phenotypes of which are well known for CSC characteristics [7, 41, 42]. However, a considerable knowledge gap remains in our understanding of the role of Orai1 in oral/oropharyngeal carcinogenesis and in the regulation of CSCs.

In the present study, we report for the first time that Orai1 expression is elevated in a stepwise manner in oral/oropharyngeal carcinogenesis and enriched in OSCC CSC populations. We further provided evidence that Orai1 promotes OSCC progression by enhancing cancer stemness *via* NFAT signaling, suggesting a novel CSC regulatory mechanism by Orai1/NFAT axis.

## RESULTS

### Orai1 is overexpressed in oral/oropharyngeal carcinogenesis

**Figure 1:**
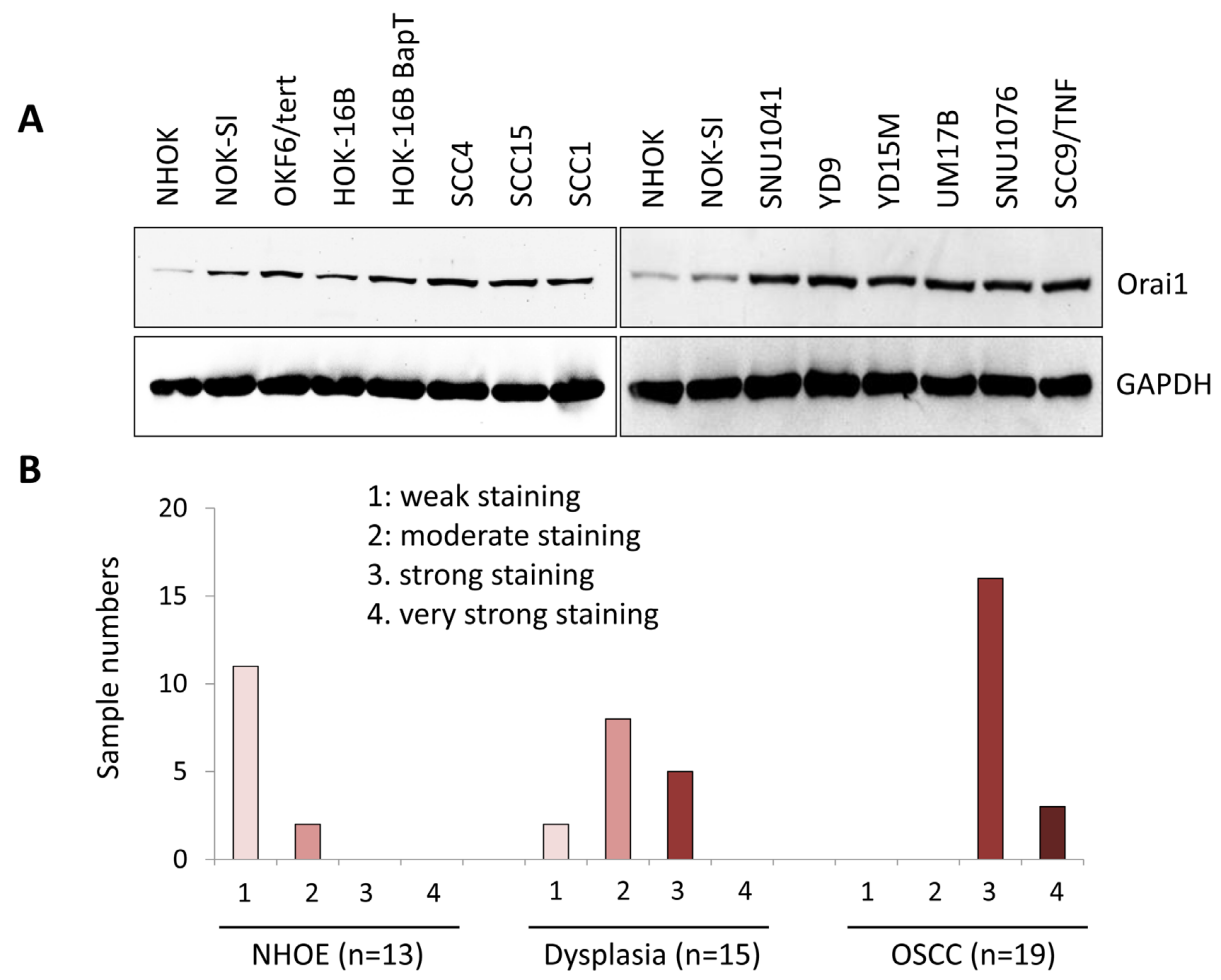

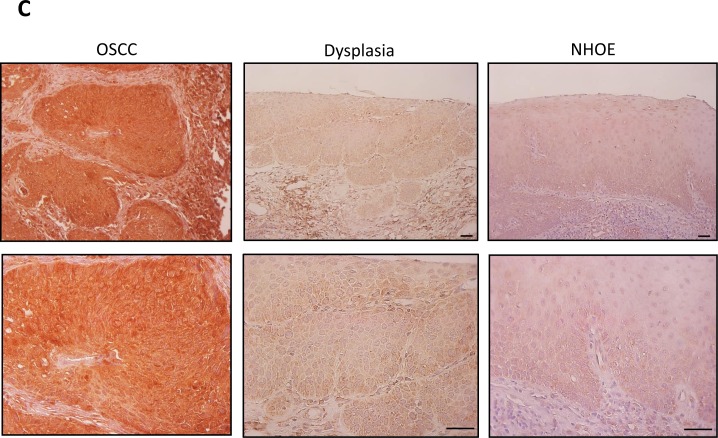
A stepwise increase of Orai1 in oral/oropharyngeal carcinogenesis **A.** Level of Orai1 protein was determined in normal human oral keratinocyte (NHOK), 3 precancerous, non-tumorigenic immortalized oral epithelial cell lines (NOK-SI, OKF6/tert, and HOK-16B) and 10 OSCC cell lines (HOK-16B-BapT, SCC4, SCC9/TNF, UM17b, and YD38) by performing Western blot. GAPDH was used as a loading control. **B.** *In vivo* Orai1 expression was determined in normal human oral epithelia (NHOE), oral dysplasia and OSCC tissues by immunohistochemical staining. **C.** Representative examples of Orai1 immunohistochemical staining in NHOE, oral dysplasia and OSCC tissues *in vivo*. Bar indicates 100 μm.

To investigate the role of Orai1 in oral/oropharyngeal carcinogenesis, we first determined the expression level of Orai1 protein in normal human oral keratinocytes (NHOK), non-tumorigenic immortalized oral epithelial cell lines (NOK-SI, OKF6/tert, and HOK-16B), and OSCC cell lines (HOK-16B-BapT, SCC4, SCC15, SCC1, SNU1041, YD9, YD15M, UM17B, SNU1076 and SCC9/TNF) by western blot analysis. All of the OSCC cell lines expressed higher level of Orai1 protein compared to the tested immoralized cell lines (Figure 1A). All immoralized cell lines showed higher expression of Orai1 protein compared to NHOK (Figure 1A). Our findings suggested a stepwise increase of Orai1 expression during oral/oropharyngeal carcinogenesis. To extend our findings, immunohistochemical (IHC) staining for Orai1 was performed using normal human oral epithelia (NHOE), oral dysplasia, and OSCC tissues. The results of *in vivo* Orai1 staining are summarized in Figure 1B, and a typical Orai1 staining observation in NHOE, dysplasia and OSCC tissue is shown in Figure 1C. In 13 NHOE, weak Orai1 staining was detected in 11 cases (84.6%), and moderate staining detected in 2 cases (15.4%). Of 15 dysplastic tissues, weak staining was detected in 2 cases (13.3%), moderate staining detected in 8 cases (53.3%), and strong staining detected in 5 cases (33.3%). In 19 OSCC samples, 16 cases (84.2%) demonstrated strong staining and 3 cases (15.8%) with very strong staining. Mean IHC scores for Orai1 in NHOE, dysplasia, and OSCC were 1.15, 2.2, and 3.16, respectively, showing statistical significant difference (*P* < 0.0001 between NHOE and dysplasia; *P* < 0.0001 between dysplasia and OSCC). Orai1 was present predominantly in the plasma membrane, with diffused staining in both the cytoplasm and nucleus (Figure 1C). Using laser capture microdissection (LCM), we determined the level of Orai1 mRNA in dysplasia and OSCC tissues and found that Orai1 mRNA is also increased in OSCC compared to dysplastic tissues (Supplementary Figure 1). Taken together, our findings clearly indicate a stepwise elevation of Orai1 protein during oral/oropharyngeal carcinogenesis, suggesting an important role of Orai1 in the progression of OSCC.

### Orai1 is required for tumorigenicity of OSCC

**Figure 2:**
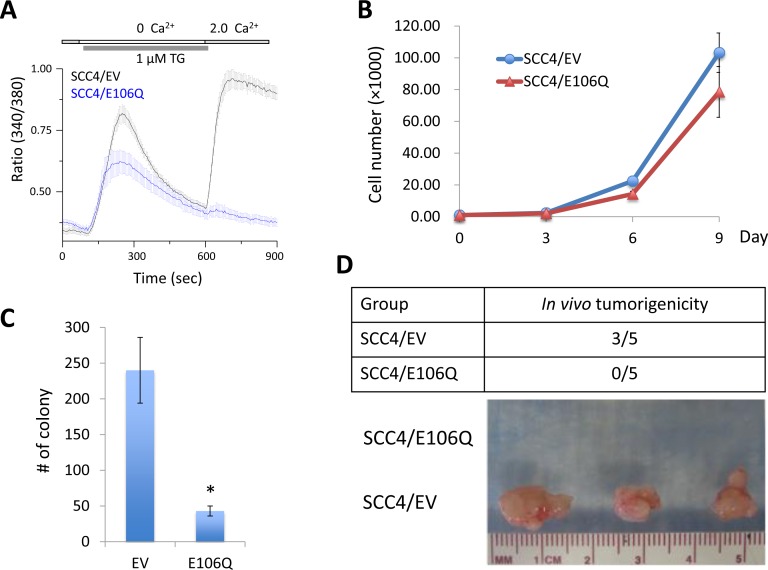
The dominant negative Orai1 mutant (E106Q) suppresses tumorigenicity of OSCC *in vivo* **A.** SCC4 cells were infected with retroviruses containing E106Q or empty vector (EV) as a control. Intracellular Ca^2+^ imaging assay was performed to confirm the inactivation of Orai1 function. Intracellular Ca^2+^ stores were depleted with 1 μM TG in the absence of extracellular Ca^2+^, followed by re-addition of 2 mM Ca^2+^. Ca^2+^ influx was analyzed by single-cell video imaging of Fura2-labeled, GFP+ cells. More than 30 GFP+ cells were analyzed in each experiment. **B.** Effect of E106Q on cell proliferation of SCC4 was determined by cell counting. Data are means ± SD of triplicate experiments. **C.** Effect of E106Q on anchorage independent growth of SCC4 was determined by soft agar assay. Data are means ± SD of triplicate experiments. *P < 0.001 by two-tailed Student's *t* test. **D.** Effect of E106Q on *in vivo* tumorigenicity of SCC4 was determined by xenograft tumor assay. SCC4/EV and SCC4/E106Q were injected subcutaneously into five nude mice. Mice were killed at week 6, and tumors were surgically removed from all animals and photographed.

Many studies reported the efficacy of targeting Orai1to suppress cancer growth [30–34]. A point mutation in the negatively charged residues of Orai1 is known to function as a dominant negative mutant [26]. To investigate the role of Orai1 in OSCC growth, we inhibited Orai1 using a dominant negative Orai1 mutant (E106Q). SCC4, a human tongue squamous carcinoma cell line, was infected with retroviral vector expressing E106Q or empty vector (EV) as a control. As shown in Figure 2A, treatment of SCC4/EV with 1 μM thapsigargin (TG), an ER Ca^2+^-ATPase inhibitor, resulted in a rapid rise in intracellular Ca^2+^ level, consistent with depletion of ER Ca^2+^ stores. Subsequent addition of Ca^2+^ to the extracellular bath solution triggered another increase in the Ca^2+^ level, consistent with Ca^2+^ influx from the extracellular solution. However, SCC4/E106Q failed to show the increase in Ca^2+^ influx. Our finding indicated that E106Q successfully impaired Orai1-mediated SOCE in OSCC cells, confirming the inactivation of Orai1 (Figure 2A). E106Q slightly lowered proliferation capacity of SCC4 (Figure 2B).

Effect of Orai1 inactivation on tumorigenic potential of OSCC was then evaluated using *in vitro* anchorage independent growth and *in vivo* tumor xenograft assay. E106Q significantly reduced formation of colonies in soft agar suggesting decreased anchorage independent growth ability by Orai1 inactivation (Figure 2C). As demonstrated by xenograft tumor assay in nude mice, 3 out of 5 animals inoculated with SCC4/EV formed tumors, whereas the animals inoculated with SCC4/E106Q failed to form tumor *in vivo* (Figure 2D). These findings indicate that Orai1 is required for tumorigenicity of OSCC.

### Orai1 is required for the maintenance of CSC phenotype and increased in CSC-enriched population

**Figure 3:**
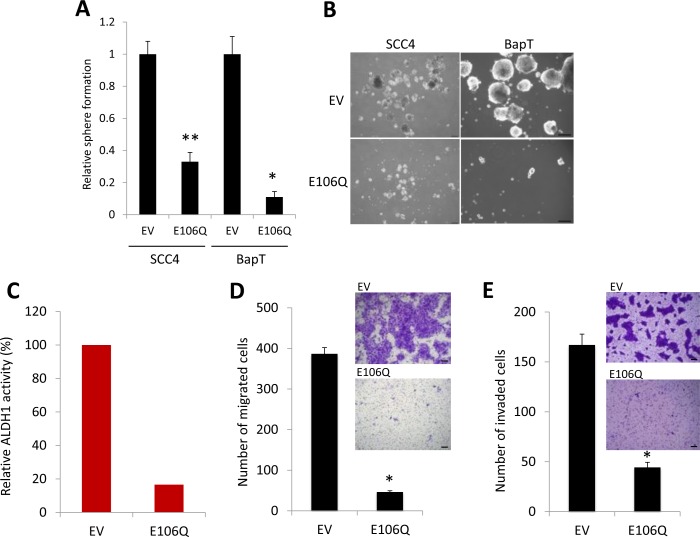

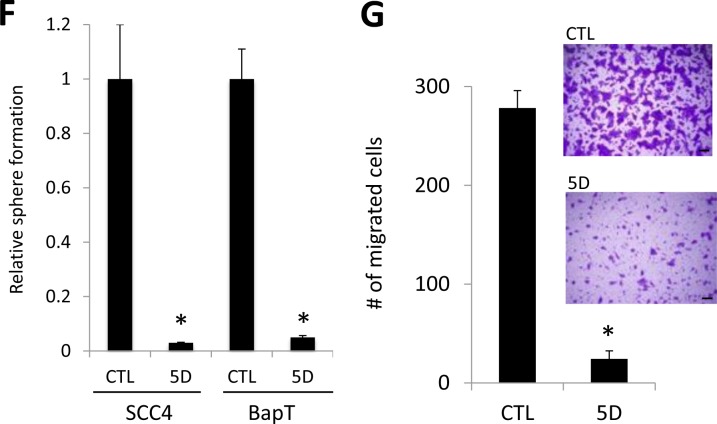
The dominant negative Orai1 mutant (E106Q) inhibits CSC phenotype **A.** Effect of E106Q on self-renewal capacity of two OSCC cell lines, SCC4 and HOK-16B BapT, was determined by tumor sphere formation assay. Data are means ± SD of triplicate experiments. *P < 0.01 and **P < 0.05 by two-tailed Student's *t* test. **B.** Representative images of tumor spheres formed by E106Q-transduced OSCC cell lines (SCC4/E106Q and BapT/E106Q) and their corresponding controls (SCC4/EV and BapT/EV). Bar indicates 100 μm. **C.** Effect of E106Q on ALDH1 activity in SCC4 was determined by Aldefluor assay. **D.** Effect of E106Q on migration ability in SCC4 was determined by transwell migration assay. Migration ability was described as number of migrated cells per field with data as mean ± SD for 3 randomly selected fields. *P < 0.01 by two-tailed Student's *t* test. Representative images of transwell migration assay are shown on the right. Bar indicates 100 μm. **E.** Effect of E106Q on invasion ability of SCC4 was determined by Matrigel invasion assay. Invasion ability was described as the number of invaded cells per field with the data as mean ± SD for 3 randomly selected fields. *P < 0.01 by two-tailed Student's *t* test. Representative images of Matrigel invasion assay are shown on the right. **F.** Effect of Orai1-specific chemical blocker, compound 5D, on self-renewal capacity of two OSCC cell lines, SCC4 and HOK-16B BapT, was determined by tumor sphere formation assay. *P < 0.01 by two-tailed Student's *t* test. **G.** Effect of compound 5D on migration ability in SCC4 was determined by transwell migration assay. *P < 0.01 by two-tailed Student's *t* test. Representative images of transwell migration assay are shown on the right.

A key feature of CSCs is self-renewal capacity, which appears to be a driving force for the initiation and maintenance of tumorigenicity [41]. Our data revealed the crucial role of Orai1 function in tumorigenicity of OSCC. To determine the role of Orai1 on CSC phenotype of OSCC, we first employed tumor sphere formation assay in which CSCs can be enriched in non-adherent tumor spheres [43]. Thus, abundance and the growth kinetics of tumor spheres are indicative of CSC content and self-renewal capacity in a given culture of heterogeneous cancer cells. We determined the effect of E106Q on self-renewal capacity of two OSCC cell lines, SCC4 and HOK-16B BapT. Tumor sphere formation assay revealed that E106Q significantly inhibits the tumor sphere forming ability of both cell lines (Figure 3A and 3B), indicating that Orai1 is indeed essential for sustaining self-renewal capacity of CSCs. ALDH1 activity is one of CSC markers and known to enrich CSCs in solid malignancies, including head and neck cancer [44]. Thus, we investigated whether Orai1 inhibition could affect CSC property by performing ALDH1 assay. The assay revealed a significant decrease in ALDH1 activity in SCC4/E106Q compared to their control SCC4/EV (Figure 3C). Because another well-known property of CSCs is their metastatic potential [41], we examined the effect of Orai1inhibiton on metastatic potential of OSCC *in vitro*. As demonstrated by a transwell migration assay (Figure 3D), E106Q markedly suppressed migration of SCC4 cells. We also found a significant inhibitory effect of E106Q on invasion ability using Matrigel invasion assay (Figure 3E). This is consistent observation reported by other [30, 45]. To extend these observations, we determined whether a chemical inhibitor of Orai1 also suppresses CSC phenotype. We treated OSCC cells with the Orai1-specific small molecular blocker, compound 5D [46] and found that the Orai1 inhibitor dramatically inhibited tumor sphere forming ability (Figure 3F) and migration (Figure 3G) at the dose of 15 μM. We observed no significant effect of compound 5D on cell proliferation at the same dose (data not shown), indicating that the inhibitory effect of compound 5D on CSC phenotype was unlikely an artifact of slower cell proliferation and a cytotoxic effect of the chemical. Similar to the effects of E106Q and compound 5D on CSC phenotype, Orai1 knockdown reduced self-renewal and migration ability of SCC4 (Supplementary Figure 2).

**Figure 4:**
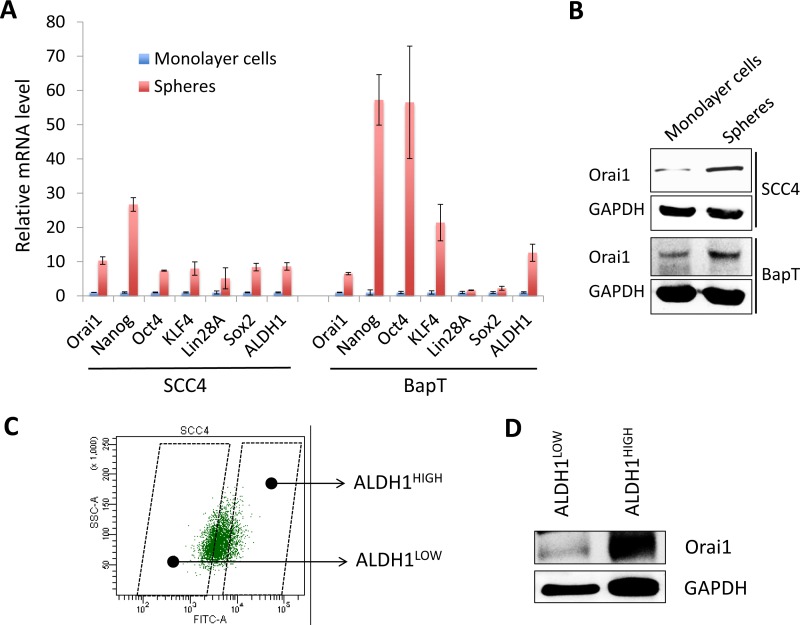
Orai1 expression is enriched in CSC populations of OSCC **A.** Expressions of Orai1, stemness transcription factors (Nanog, Oct4, KLF4, Lin28A, and Sox2) and ALDH1were assessed in tumor spheres (CSC-enriched population) and corresponding adherent monolayer cells (non-CSC population) derived from SCC4 and HOK-16B BapT by real-time qPCR. Levels of the genes were normalized with the level of GAPDH. Their levels in tumor spheres were plotted as fold induction against those in their corresponding adherent monolayer cells. **B.** Level of Orai1 protein was assessed in tumor spheres and corresponding adherent monolayer cells by Western blot analysis. **C.** Demonstration of the strategy for sorting ALDH1^HIGH^ (CSC-enriched population) and ALDH1^LOW^ (non-CSC population) cell population. ALDH1^HIGH^ and ALDH1^LOW^ cell populations were sorted from SCC4 cells by flow cytometry. **D.** Level of Orai1 protein was determined in ALDH1^HIGH^ and ALDH1^LOW^ cell population by Western blot analysis.

To further examine the importance of Orai1 in CSC, we compared Orai1 expression of CSC-enriched populations with that of non-CSC populations. Orai1 expression is highly enriched in tumor spheres compared with their corresponding adherent monolayer cells (Figure 4A and 4B). Similarly, stemness transcription factors, Nanog, Oct4, KLF4, Lin28, and Sox2 were enriched in tumor spheres (Figure 4A). We also observed robust induction of CSC marker, ALDH1, in tumor spheres (Figure 4A), confirming that tumor spheres are CSC-enriched cell population. Furthermore, we sorted the ALDH1^HIGH^ and ALDH1^LOW^ cell populations from SCC4 according to ALDH1 activity (Figure 4C) and examined Orai1 expression in these two cell populations. The ALDH1^HIGH^ population expressed higher Orai1 protein than the ALDH1^LOW^ population (Figure 4D). Overall, our data clearly indicate that Orai1 is enriched in CSC and essential for maintenance of CSC phenotype in OSCC.

### Orai1 endows non-tumorigenic oral epithelial cells with tumorigenic potential and CSC phenotype

**Figure 5:**
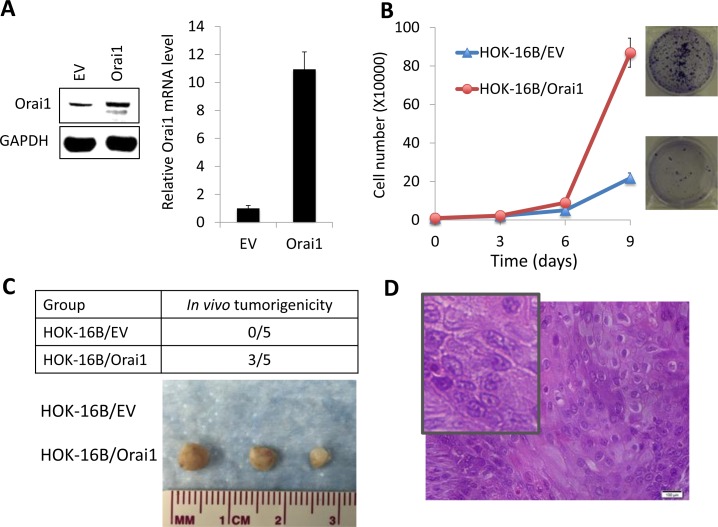
Ectopic Orai1 expression endows non-tumorigenic immortalized oral epithelial cells with tumorigenic potential *in vivo* **A.** Orai1 expression was forced in non-tumorigenic immortalized oral epithelial cells, HOK-16B, by infecting with retroviral vector expressing Orai1, and its ectopic expression was confirmed by Western blot analysis (Left) and real-time qPCR (Right). **B.** Effect of Orai1 on cell proliferation of HOK-16B was determined by cell counting. Data are means ± SD of triplicate experiments. Representative images of clonogenic assay are also shown on the right. **C.** Effect of Orai1 on *in vivo* tumorigenicity of HOK-16B was determined by xenograft tumor assay. HOK-16B/EV and HOK-16B/Orai1 were injected subcutaneously into five nude mice. Mice were killed at week 6, and tumors were surgically removed from all animals and photographed. **D.** Morphology characteristics of xenografts formed by HOK-16B/Orai1 were observed *via* hematoxylin and eosin (H&M) staining. Insert showing magnified image.

Having established that increased Orai1 is associated with OSCC progression and is necessary for CSC phenotype, we next examined whether ectopic Orai1 expression confers tumorigenic capacity and CSC phenotype on non-tumorigenic immortalized oral epithelial cells. As shown in Figure 5A, we overexpressed Orai1 in non-tumorigenic immortalized oral epithelial cells, HOK-16B, using the lentiviral vector expressing Orai1 or empty vector (EV) as a control. We first examined the effect of Orai1 on cell proliferation and found that Orai1 overexpression led to robust increase in proliferation capacity of HOK-16B *in vitro* (Figure 5B). To further examine the effect of Orai1 on tumor growth *in vivo*, we injected the cells into nude mice and observed tumor formation. Three out of 5 mice injected with HOK-16B/Orai1 developed tumor, whereas, as expected, the mice injected with HOK-16B/EV failed to form tumor (Figure 5C). Histological examination of tumors grown in mice injected with HOK-16B/Orai1 revealed the presence of malignant cells, with hyperchromatic nuclei, prominent nucleoli, and active mitosis (Figure 5D), indicating that Orai1 conferred tumorigenicity to the non-tumorigenic cells.

**Figure 6:**
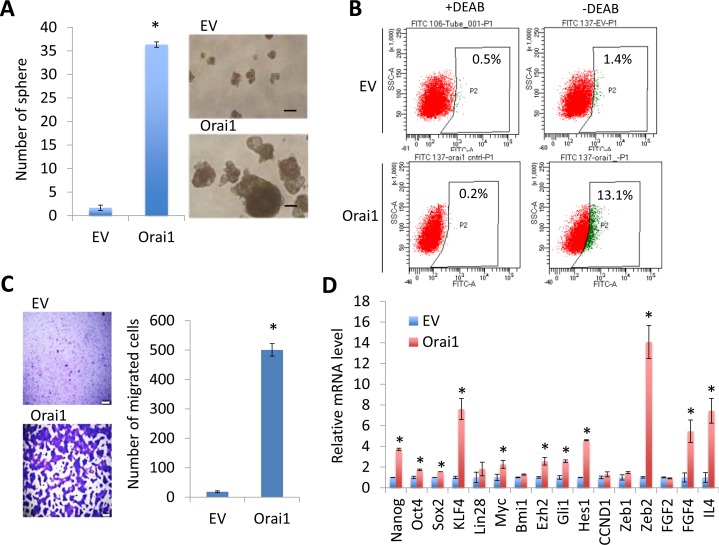
Ectopic Orai1 expression promotes CSC phenotype in non-tumorigenic immortalized oral epithelial cells **A.** Effect of Orai1 on self-renewal capacity of HOK-16B was determined by tumor sphere formation assay. *P < 0.01 by two-tailed Student's *t* test. Representative image of tumor spheres formed by HOK-16B/EV and HOK-16B/Orai1 are shown on the right. Bar indicates 100 μm. **B.** Effect of Orai1 on ALDH1 activity in HOK-16B was determined by Aldefluor assay. Cells were labeled with Aldefluor combined with or without the ALDH1 inhibitor DEAB and analyzed by flow cytometry. The gate for ALDH1 + cells is determined in relation to the DEAB control (+DEAB) and shows the brightly fluorescent ALDH1 population *versus* the side scatter, a population that is absent/decreased in the presence of DEAB. The number shown in each panel reflects the percentage of ALDH1+ cells in each cell type. **C.** Effect of Orai1 on migration ability in HOK-16B was determined by transwell migration assay. *P < 0.001 by two-tailed Student's *t* test. Representative images of transwell migration assay are shown on the left. **D.** Effects of Orai1 on stemness transcription factors (Nanog, Oct4, Sox2, KLF4, Lin28, Myc, and Bmi1) and CSC-related genes (Ezh2, Gli1, Hes1, CCND1, Zeb1, Zeb2, FDF2, FGF4, and IL4) in HOK-16B were determined by real-time qPCR. Their levels in HOK-16B/Orai1 were plotted as fold induction against those in HOK-16B/EV. *P < 0.05 by two-tailed Student's *t* test.

Next, we investigated the effect of Orai1 on CSC phenotype in HOK-16B. Ectopic Orai1 expression resulted in robust induction in tumor sphere formation, indicating the acquisition of self-renewal capacity by Orai1 (Figure 6A). The flow analysis revealed a significant increase in ALDH1+ cell population in HOK-16B/Orai1 compared to HOK-16B/EV (13.1% *vs*. 1.4%; Figure 6B). As demonstrated by a transwell migration assay (Figure 6C), HOK-16B/Orai1 migrated significantly faster than HOK-16B/EV. The effect of Orai1 on CSC phenotype was further validated by qPCR analysis of stemness transcription factors and CSC-related genes (Figure 6D). Orai1 increased stemness transcription factors (i.e., Nanog, Oct4, Sox2, KLF4, and Myc) and CSC-related genes (i.e., Ezh2, Gli1, Hes1, Zeb2, FGF4, and IL4). Our findings indicate that Orai1 indeed conferred CSC phenotype on the non-tumorigenic oral epithelial cells.

### NFAT signaling is indispensable for Orai1-induced CSC phenotype

**Figure 7:**
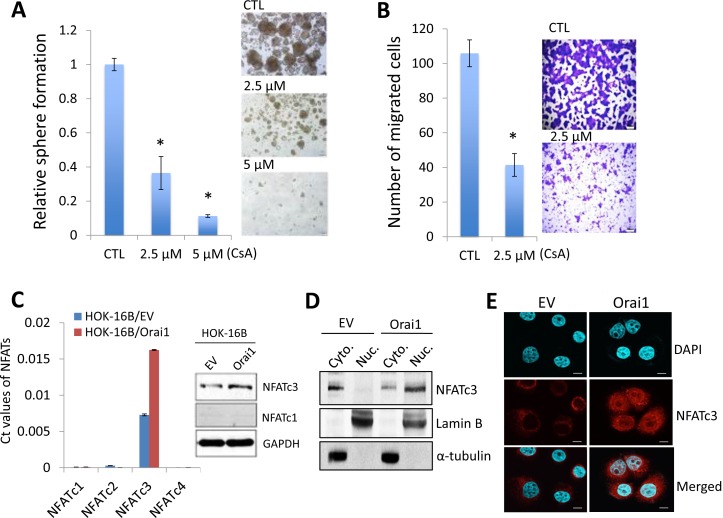

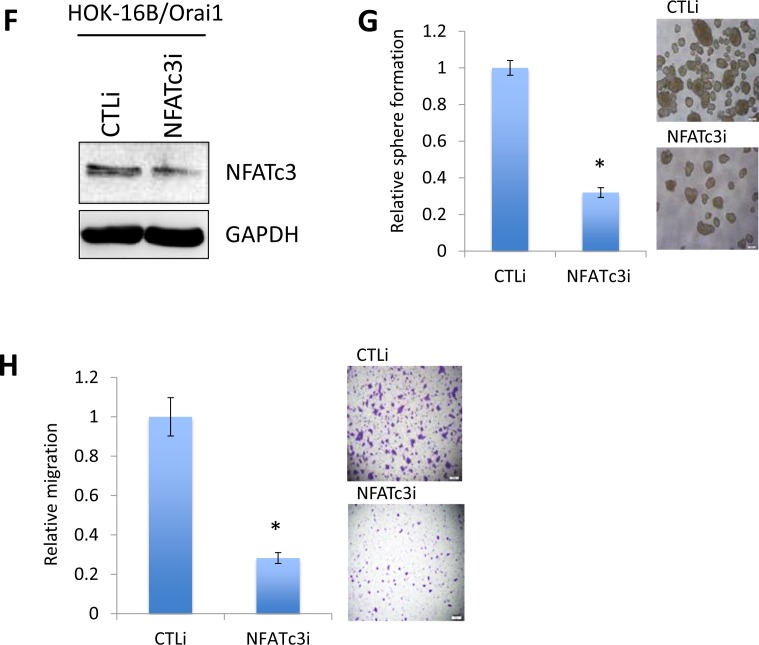
NFAT signaling is required for Orai1-induced CSC phenotype **A.** Effect of NFAT antagonist, cyclosporine A (CsA), on self-renewal capacity of HOK-16B/Orai1 was determined by tumor sphere formation assay. *P < 0.05 by two-tailed Student's *t* test. Representative images of tumor spheres formed by HOK-16B/Orai1 exposed to the NFAT inhibitor are shown on the right. **B.** Effect of the NFAT inhibitor on migration ability in HOK-16B/Orai1 was determined by transwell migration assay. *P < 0.05 by two-tailed Student's *t* test. Representative images of transwell migration assay are shown on the right. **C.** Effect of Orai1on the expression of NFAT isoforms (NFATc1, NFATc2, NFATc3, and NFATc4) in HOK-16B was determined by real-time qPCR (left) and Western blot analysis (right). The Ct values for 4 NFAT isoforms were normalized by the CT value of GAPDH. **D.** Effect of Orai1 on the intracellular localization of NFATc3 was determined by Western blot analysis using the cytoplasmic (Cyto.) and nuclear (Nuc.) extracts. Lamin B is a nuclear protein. α-tubulin is a cytoplasmic protein. **E.** Effect of Orai1 on the intracellular localization of NFATc3 was determined by confocal laser scanning microscopy. After cell permeabilization and blocking, cells were probed with NFATc3 primary antibody overnight, then with Alexa Fluor 594 dye-conjugated secondary antibody and DAPI (blue-green) for confocal laser scanning. HOK-16B/EV has NFATc3 immunofluorescence staining (red) mainly in the cytoplasm while HOK-16B/Orai1 has stronger staining both in the cytoplasm and the nucleus, which indicates increased NFATc3 expression as well as dominant nuclear translocation. Representative images were taken at 60x. **F.** Endogenous NFATc3 was knocked down in HOK-16B/Orai1 using siRNA against NFATc3 (NFATc3i). The cells transfected with control siRNA (CTLi) were included for comparison. Knockdown of NFATc3 was confirmed by Western blot analysis. **G.** NFATc3 knockdown effect on self-renewal capacity of HOK-16B/Orai1 was determined by tumor sphere formation assay. Representative images of tumor spheres formed by HOK-16B/Orai1 transfected with control siRNA (HOK-16B/Orai1/CTLi) and NFATc3 siRNA (HOK-16B/Orai1/NFATc3i) are shown on the right. **H.** NFATc3 knockdown effect on migration ability in HOK-16B/Orai1 was determined by transwell migration assay. *P < 0.05 by two-tailed Student's *t* test. Representative images of transwell migration assay are shown on the right. Bar indicates 100 μm.

It is well documented that Orai1-mediated SOCE activates downstream responses including NFAT signaling pathway [27, 28]. Emerging evidence has suggested that NFAT signaling plays an important role in tumorigenesis [47]. Thus, we investigated whether Orai1 promotes CSC phenotype through NFAT signaling pathway. We treated HOK-16B/Orai1 with the NFAT antagonist, cyclosporine A (CsA) [48], and subsequently performed the assays for CSC properties. The NFAT inhibitor significantly inhibited self-renewal (Figure 7A) and migration (Figure 7B) of HOK-16B/Orai1. The NFAT inhibitor also suppressed self-renewal and migration in OSCC cells (Supplementary Figure 3). Our data indicate that NFAT signaling is required for Orai1-induced CSC phenotype.

Four isoforms of NFAT, NFATc1, NFATc2, NFATc3, and NFATc4, were identified [49]. To determine which of the NFAT isoforms are involved in Orai1-induced CSC phenotype, we first examined the expressions of the isoforms in HOK-16B/EV and HOK-16B/Orai1. Among these four members, we found that NFATc3 was the dominant isoform and increased by Orai1 in HOK-16B (Figure 7C). Moreover, knockdown of Orai1 reduced the expression of NFATc3 (Supplementary Figure 2A). We also found that NFATc3 is the dominant isoform in various OSCC cell lines (data not shown). Notably, NFATc3 was primary found in the cytoplasm of HOK-16B/EV but accumulated in the nucleus of HOK-16B/Orai1 (Figure 7D and 7E), indicative of NFATc3 activation by Orai1 [47].

To further assess the functional role of NFATc3 in Orai1-induced CSC phenotype, we knocked down NFATc3 using siRNA in HOK-16B/Orai1 (Figure 7F). Knockdown of NFATc3 showed significant suppressive effect on tumor sphere formation (Figure 7G) and migration (Figure 7H) in HOK-16B/Orai1 cells. Consistent with this, we also demonstrated that silencing of NFATc3 resulted in significant suppression of tumor sphere formation and migration ability in OSCC cells (Supplementary Figure 4). Our findings indicate that Orai1 promotes CSC phenotype through NFATc3, suggesting the role of Orai1/NFAT axis in CSC regulation.

## DISCUSSION

In this study, we demonstrate for the first time that Orai1 is a novel molecular regulator of tumorigenicity and stemness of OSCC. Orai1 expression is elevated in a stepwise manner during oral/oropharyngeal carcinogenesis and enriched in CSC populations. Ectopic Orai1 expression confers *in vivo* tumorigenic capacity and CSC phenotype on non-tumorigenic immortalized oral epithelial cells. Moreover, inhibition of Orai1 suppresses tumorigenicity and CSC phenotype in OSCC. We also provide the evidence that Orai1 enhances CSC phenotype through NFAT signaling, indicating the importance of Orai1/NFAT axis in oral CSC. Therefore, our study highlights the functional significance of Orai1 signaling in malignant progression of OSCC by enriching cancer stemness.

Emerging role of Orai1 in human cancer has been reported. Upregulation of Orai1 expression was observed in various human cancers, including esophageal cancer [34]. A high expression of Orai1 protein is also strongly linked to poor prognosis and aggressive behavior of human cancers. However, no information is available regarding Orai1 expression during oral/oropharyngeal carcinogenesis. Our results showed that Orai1 is highly expressed in OSCC compared to precancerous and normal tissues *in vivo*. Moreover, precancerous oral epithelial cells express higher Orai1 protein than normal oral epithelial cells, suggesting that Orai1 plays an important role in cancer progression. To our knowledge, our finding is the first report showing a stepwise elevation of Orai1 in multistep carcinogenesis *in vivo* and further oncogenic transformation of immortalized cells by Orai1 overexpression. Consistent with previous observation [34], our immunohistochemistry study confirmed localization of Orai1 in the plasma membrane. We also observed diffused staining of Orai1 in both the cytoplasm and nucleus. Since the tissue sections were obtained from 3 dimensional tissue structures, there could be some overlap with another cell layer positioned in different orientation compared to monolayer-cultured cells. Indeed, we were able to detect predominant Orai1 staining to the plasma membrane in the monolayer-cultured cells of HOK-16B/Orai1 (data not shown). Our study clearly demonstrated that Orai1 is required for tumorigenicity of OSCC *in vivo*. Inactivation of Orai1 by the dominant negative Orai1 mutant not only suppressed SOCE, but also abolished *in vivo* tumorigenic potential of OSCC. Conversely, ectopic Orai1 expression further transformed non-tumorigenic oral epithelial cells to tumorigenic cells. This finding is consistent with the results of a previous study reporting that Orai1 inhibition led to significant decreases in cancer growth *in vivo* and *in vitro* [32–34]. Our findings support the hypothesis that Orai1 is a novel molecular determinant of oral/oropharyngeal cancer progression.

Self-renewal is the critical characteristic by which CSCs regenerate themselves, suggesting the driving force of tumorigenesis. CSCs are viewed as the seed of cancer and hence as effective target of anti-cancer therapies. Therefore, findings from our studies are of paramount important for the development of more effective cancer therapies. By phenotypic and functional analysis, we demonstrate that Orai1 is an important regulator of CSC phenotype in OSCC. Orai1 is highly expressed in CSC-enriched cell populations, such as tumor spheres and ALDH1^HIGH^ population of OSCC. Furthermore, Orai1 endowed non-tumorigenic immortalized oral epithelial cells with self-renewal and concomitantly increased stemness transcription factors, *i.e*., Nanog, Oct4, Sox2, KLF4, and Myc. Orai1 also promoted other CSC properties, ALDH1 activity and migration. Ectopic Orai1 expression markedly increases ALDH1+ cell population in the non-tumorigenic oral epithelial cells. ALDH1 has been found to be a marker for stem cells in different types of cancer, including OSCC [50, 51]. ALDH1+ cancer cells displayed higher self-renewal, migration, and tumorigenic potential than ALDH1- cells [50, 52, 53]. Orai1 also markedly increases motility of the non-tumorigenic cells. Our finding is consistent with previous reports showing the importance of Orai1 in cell migration. Knockdown of Orai1 in invasive breast cancer cell lines decreased cell migration, whereas its overexpression promoted cellular motility [36]. However, underlying mechanism by which Orai1 enhances oral epithelial cell migration has not been understood. Therefore, effects of Orai1 on epithelial-to-mesenchymal transition (EMT) and metastasis-related gene expression should be warranted to investigate [54]. In multiple OSCC cell lines, inhibition of Orai1 led to suppression of such CSC phenotype. We conclude that Orai1 increases not only the number of CSCs, but also CSC properties. Thus, we hypothesize that Orai1 promotes malignant progression of OSCC by enriching CSC phenotype. Therefore, Orai1 could be an effective therapeutic target for OSCC.

We also found that Orai1 regulates the expression of several important CSC-related genes, *i.e., Ezh2, Gli1, Hes1, Zeb2, FGF4, and IL4*. Studies have shown the important role of these genes in acquisition and maintenance of CSC phenotype. For instance, our previous study demonstrated that Hes1 is upregulated in OSCC, and the suppression of Hes1 in oral cancer cells inhibits self-renewal capacity of OSCC, suggesting the important role of Hes1 in OSCC CSC [55]. In addition, other reports also revealed the crucial roles of Hes1 in the maintenance of CSC properties, such as metastasis, chemotherapy resistance, and EMT [56]. Zeb1 and Zeb2 are significantly increased in head and neck CSCs compared to non-CSCs [57]. Knockdown of Zeb1 and Zeb2 in head and neck cancer cells decreased their CSC properties such as self-renewal capacity, the expression of stemness markers, and drug resistance. Moreover, their suppression inhibited *in vivo* tumor growth and the rate of metastasis to distant site [57]. Conversely, co-overexpression of Zeb1 and Zeb2 enhanced sphere-forming ability of head and neck cancer cells [57]. FGF4 is shown to play a key role in maintaining self-renewal capacity of normal and cancer stem cells [58, 59]. FGF4 promoted self-renewal of CSC-enriched population [59].

Many studies demonstrated that cytokines increased CSC population and phenotype [55, 60–63]. We also reported that inflammatory cytokine enhanced CSC phenotype of OSCC [55]. Recently, to investigate the roles of cytokines in CSCs of OSCC, we profiled expression of 25 cytokines and found that IL4 was highly increased in tumor spheres compared to corresponding adherent monolayer cells (manuscript in preparation). IL4 is overexpressed in different types of cancer including breast, ovarian, colon, lung, and thyroid [64]. Moreover, recent studies revealed crucial role of IL4 in the maintenance of CSC [61, 65, 66]. We found that IL4 expression is upregulated by Orai1 and downregulated by inhibition of Orai1. Since IL4 is a well-known target of NFAT, the major downstream effector molecule of Orai1 [67, 68], we speculate a possible role of Orai1-NFAT-cytokine(s) axis in CSC regulation; however, further investigation will be necessary to examine the functional roles of cytokines, *e.g.,* IL4, in CSC regulation by Orai1/NFAT axis.

Orai1-mediated SOCE activates NFAT, a family of transcription factors composed of four members, NFATc1, NFATc2, NFATc3, and NFATc4 [27, 28, 49]. Emerging evidence has suggested that NFAT signaling plays an important role in tumorigenesis by regulating various target genes involved in cancer development [47]. For instance, NFATc1 induced malignant cell growth phenotype in pancreatic cancer cells by upregulating Myc [69] and promoted metastasis of mammalian cancer cells *via* MMP-2 upregulation [70–72]. NFATc2 was overexpressed in multiple cancer types [71, 72], and its depletion suppressed migration/invasion of cancer cells [71]. However, the role of NFAT proteins in OSCC has not been documented. Treatment of OSCC cells with NFAT inhibitor suppressed self-renewal and migration. Similar effect of the NFAT inhibitor was also observed in HOK-16B/Orai1, suggesting that NFAT is required for Orai1-induced CSC phenotype. Furthermore, our studies revealed that NFATc3 is dominant isoform in oral epithelial cells and activated by Orai1 overexpression. Silencing NFATc3 in HOK-16B/Orai1 led to suppression of CSC phenotype, suggesting the funtional role of Orai1/NFAT axis in the regualtion of CSC.

There is emerging evidence suggesting that spatio-temporal regulation calcium signaling (calcium oscillation) is more important than global changes in cytosolic calcium concentration in the context of tumor invasion, growth and cancer cell stemness [34, 73, 74]. Calcium oscillation is the result of SOCE. Orai1 is the dominant isoform in OSCC and enriched in OSCC CSCs. Although our work has not demonstrated the calcium oscillation change in Orai1-overexpressing cells, we have shown that inhibiting Orai1 channel function resulted in complete shutdown of the calcium oscillation.

In conclusion, Orai1 is a novel molecular regulator of tumorigenicity and stemness of OSCC. Orai1 enhances CSC phenotype through NFAT signaling. Thus, the Orai1/NFAT axis could be an important therapeutic target in OSCC. Since Orai1 is readily inhibited by small molecular inhibitor [46], targeting Orai1 signaling may be a plausible therapeutic modality against cancer.

## MATERIALS AND METHODS

### Cell culture and reagents

Primary normal human oral keratinocytes (NHOK) were prepared from oral mucosa and cultured in Keratinocyte Growth Medium (KGM, Lonza) as described previously [75]. Three non-tumorigenic immortalized oral epithelial cell lines, NOK-SI [76], OKF6/tert [77], and HOK-16B [75], were also cultured in KGM. Ten human OSCC cell lines, HOK-16B BapT, SCC4 [78], SCC15, SCC1, SNU1041, SNU1076, SCC9/TNF [55], YD9 [79], YD15M, and UM17b [80], were cultured in DMEM/Ham's F12 (Invitrogen) supplemented with 10% FBS (Gemini Bioproducts) and 0.4 μg/ml hydrocortisone (Sigma-Aldrich). Orai1 chemical blocker, Compound 5D [46], was obtained from Dr. Yousang Gwack (UCLA David Geffen School of Medicine). Antagonist of NFAT signaling, cyclosporine A (CsA), was purchased from Sigma-Aldrich.

### Western blotting

Western blotting was performed as described previously [55]. We used the following primary antibodies for our study: Orai1 (AB9868; EMD Millipore), NFATc3 (sc-8405; Santa Cruz Biotech), NFATc1 (8032S; Cell Signaling Tech), Lamin B1 (sc-20682; Santa Cruz Biotech), α-tubulin (T9026; Sigma) and GAPDH (FL-335; Santa Cruz Biotech). Cytoplasmic and nuclear proteins were isolated using the NE-PER^®^ Nuclear and Cytoplasmic Extraction Reagents kit (Pierce, Rockford) following the manufacturer's instructions.

### Immunohistochemistry

Tissue specimens that were previously collected for diagnostic purposes were obtained from the Oral Pathology Diagnostic Laboratory at the UCLA School of Dentistry. All tissue specimens were collected and processed according to the guidelines of the University of California at Los Angeles Institutional Review Board. Immunohistochemical staining was performed as described previously [81]. The optimal concentration (1:100) of Orai1 antibody (AB9868; EMD Millipore) was first established using serially diluted primary antibody along with IgG as a negative control. Two independent examiners scored the immunohistochemical expression of Orai1 protein. The level of Orai1 staining pattern was scored into four subgroups: (1) weak; (2) moderate; and (3) strong (+++); (4) very strong.

### Confocal laser scanning microscopy

Five thousand cells were seeded on the 4 chamber slides (Thermo Fisher Scientific) one day prior to the immunofluorescence staining. After cell permeabilization and blocking, cells were probed with NFATc3 primary antibody overnight, then with Alexa Fluor 594 dye-conjugated secondary antibody and DAPI (blue-green) for confocal laser scanning. Confocal laser scanning microscopy was performed using Fluoview FV10i Confocal Microscope (Olympus), and images were captured with 60x oil objective under different gain settings. The laser diode 559 nm was used to capture NFATc3 staining, and the diode 405 nm laser was used to capture DAPI nuclear stain. Image acquisition and further adjustment of brightness was performed using Olympus Fluoview Ver. 4.2a. Fluorescent images of cells were taken as single channel images then converted to overlay images and all images were saved in TIFF format.

### Ectopic expression of dominant negative Orai1 E106Q mutant and wild-type Orai1

The retroviral pMSCV-CITE-eGFP-Puro vectors encoding ORAI1^WT^ and ORAI1^E106Q^ [26] were used to prepare viruses as described previously [82]. These vectors were transfected into GP2-293 universal packaging cells (Clonetech, Mountain View, CA, USA) along with pVSV-G envelope plasmid using lipofectamine 2000 (Life Technologies). Detailed methods of retrovirus production and infection can be found in our previous publications [83]. Infected cells were selected with 0.5μg/ml puromycin for two weeks and used for experiments.

### SOCE assay (Single-cell Ca^2+^ imaging)

Cells were plated on UV-sterilized coverslips one day prior to imaging. Next day, cells were loaded with 1 mM Fura 2-AM for 45 min at 25°C. Intracellular [Ca^2+^]_i_ measurements were performed using essentially the same methods as previously described [84].

### Anchorage-independent growth

To determine colony-forming efficiency in semi-solid medium, 1 × 10^4^ cells were plated in culture medium containing 0.3% agarose over a base layer of serum-free medium containing 0.5% agarose. The assay was performed as described previously [55].

### *In vivo* xenograft tumor assay

Five-ten million cells were subcutaneously injected into the flank of immunocompromised mice (strain *nu/nu*, Charles River Laboratories). The animal study was performed according to the protocol approved by UCLA Animal Research Committee. The kinetics of tumor growth was determined by measuring the volume in three perpendicular axes of the nodules using micro-scaled calipers.

### Tumor sphere formation assay

Three thousand cells were grown in 3 ml of serum-free DMEM/F12 media supplemented with 1:50 B27 (Invitrogen), 20 ng/mL EGF, 20 ng/mL, 10μg/mL insulin, penicillin, streptomycin, and amphotericin B in Ultra-Low Attachment 6-well Plates (Corning) for 6-10 days [55].

### Quantitative real-time PCR (qPCR)

cDNA was synthesized from 5 μg of total RNA using SuperScript first-strand synthesis system (Invitrogen). We used 1 μl cDNA for qPCR amplification using SYBR Green I Master mix (Roche) and LightCycler 480 II (Roche). The primer sequences were obtained from the Universal Probe Library (Roche), and the sequences can be available upon request. Second derivative Cq value determination method was used to compare fold-differences according to the manufacturer's instructions.

### ALDH1 assay

Using Aldehyde Dehydrogenase-Based Cell Detction Kit (STEMCELL), ALDH enzymatic activity was determined. Total of 1 x10^6^ cells were re-suspended in the ALDEFLUOR Assay Buffer in the volume of 1ml. Fluorescent nontoxic ALDEFLUOR Reagent BODIPY^TM^ (1.25μl) was added as a substrate to measure ALDH enzymatic activity in intact cells. Immediately after adding the substrate reagent, 0.5ml of the cell suspension was transferred into the control tube which contains specific inhibitor for ALDH, diethylaminobenzaldehyde (DEAB) for calculating background fluorescence. Then, cells were incubated at 35°C for 30 minutes and fluorescence data acquisition was made by using a BD FACScan flow cytometer (BD Biosciences).

### Migration and invasion assays

Cell migration was measured using transwell chambers with polycarbonate membranes (Corning) as described in our previous publication [55]. Cell invasion was measured using Matrigel Basement Membrane Matrix (BD Biosciences), according to the method as described in manufacture protocol.

### Knockdown of NFATc3

NFATc3 was knocked down by duplex siRNA targeting NFATc3 or the control, scrambled siRNA (Santa Cruz Biotech), which was introduced using Lipofectamine RNAiMAX (Invitrogen). Cells (2 × 10^5^) were plated in 60-mm dishes and transfected with 15 μg siRNA. The cultures were harvested after two days post-transfection for expression and functional analyses.

## SUPPLEMENTARY MATERIAL FIGURES



## Abbreviations

OSCC: Oral/oropharyngeal squamous cell carcinomas
CSCs: Cancer stem cells
SOCE: store-operated Ca2+ entry
NHOE: Normal human oral epithelia
ALDH1: Aldehyde dehydrogenase 1
NFAT: nuclear factor of activated T cells
IL4: Interleukin 4
siRNA: small interfering RNA
IHC: immunohistochemistry
