# Supplementary material for: Orai1 promotes tumor progression by enhancing cancer stemness via NFAT signaling in oral/oropharyngeal squamous cell carcinoma
Source: Oncotarget. 2016 Jun 1;7(28):43239–55. doi: 10.18632/oncotarget.9755 (PMC5190020; doi:10.18632/oncotarget.9755)
Supplement: Supplementary file 1 [file oncotarget-07-43239-s001.pdf]

Orai1 promotes tumor progression by enhancing cancer stemness *via* NFAT signaling in oral/oropharyngeal squamous cell carcinoma

Supplementary Material

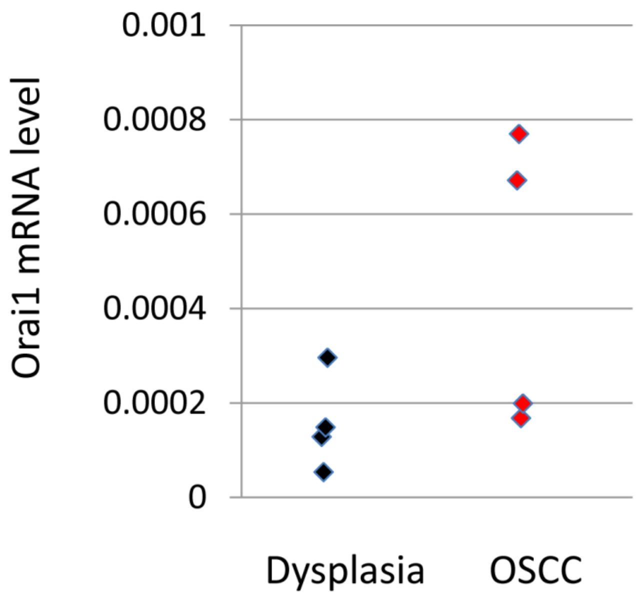

Supplementary Figure 1. Increased Orai1 mRNA in OSCC. OSCC and dysplastic cells were microdissected from oral cancer tissue and oral dysplastic tissue, respectively. The levels of Orai1 mRNA were measured by qRT-PCR and normalized with the expression of GAPDH.

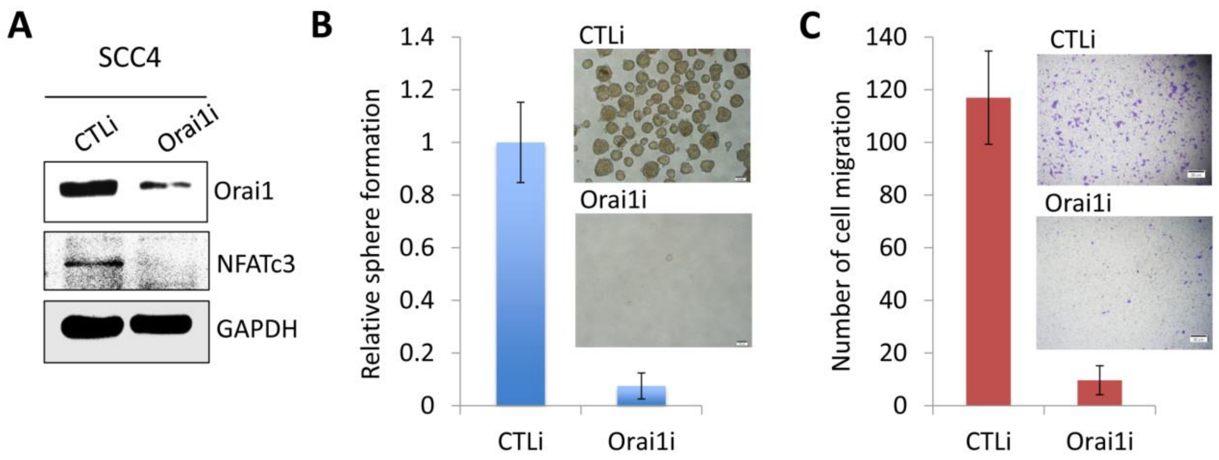

Supplementary Figure 2. Effect of Orai1 knockdown on self-renewal and migration of OSCC. (A) Endogenous Orai1 was knocked down in SCC4 using siRNA against Orai1 (Orai1i: Santa Cruz). The cells transfected with control siRNA (CTLi) were included for comparison. (B) Effect of Orai1 knockdown on self-renewal capacity of SCC4 was determined by tumor sphere formation assay. Data are means ± SD of triplicate experiments. (C) Effect of Orai1 knockdown on migration ability of SCC4 was determined by transwell chamber. Migration ability was described as number of migrated cells per field with data as mean ± SD for 3 randomly selected fields. Bar indicates 100 μm.

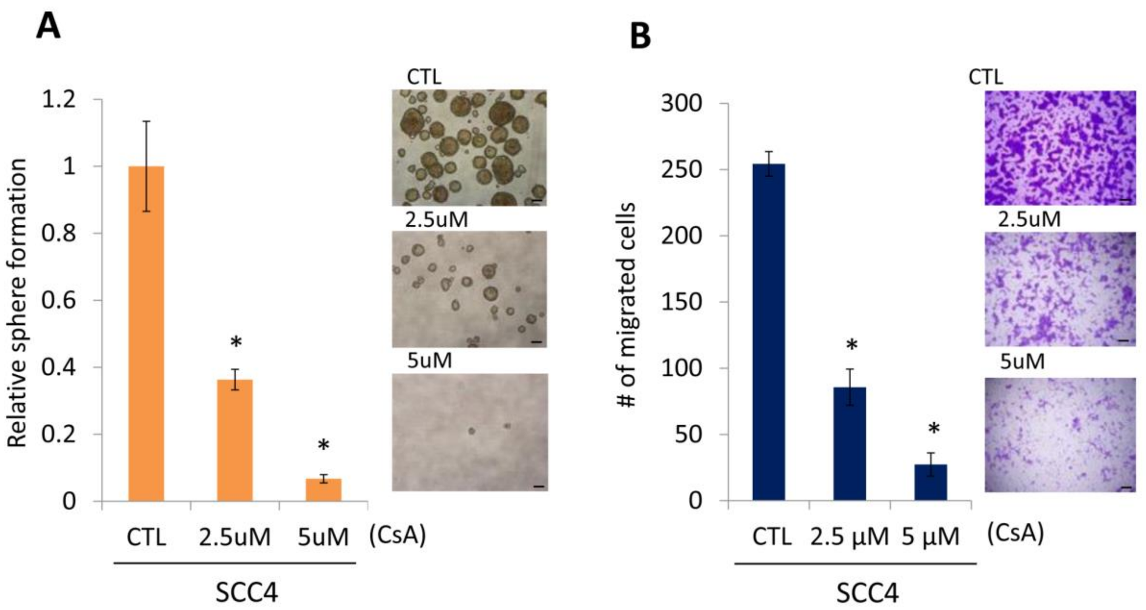

Supplementary Figure 3. Inactivation of NFAT suppresses CSC phenotype in OSCC. (A) Effect of NFAT inhibitor CsA on self-renewal capacity of SCC4 was determined by tumor sphere formation assay. **P* < 0.01 by two-tailed Student's *t* test. Representative images of tumor spheres formed by SCC4 exposed to CsA are shown on the right. (B) Effect of NFAT inhibitor CsA on migration ability in SCC4 was determined by transwell migration assay. **P* < 0.01 by two-tailed Student's *t* test. Representative images of transwell migration assay are shown on the right. Bar indicates 100 µm.

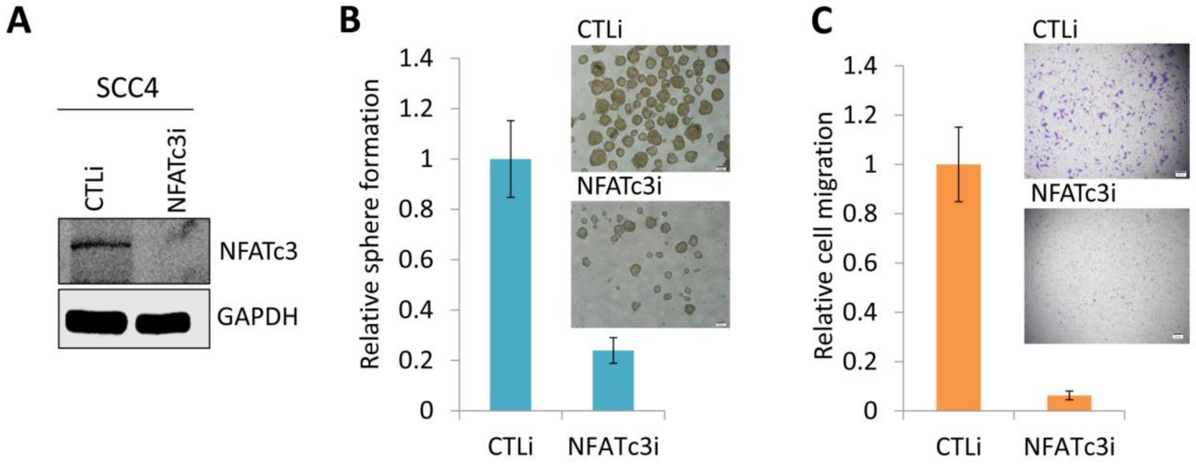

Supplementary Figure 4. Effect of NFATc3 on CSC phenotype in OSCC. (A) Endogenous NFATc3 was knocked down in SCC4 using siRNA against NFATc3 (NFATc3i). The cells transfected with control siRNA (CTLi) were included for comparison. (B) Effect of NFATc3 knockdown on self-renewal capacity of SCC4 was determined by tumor sphere formation assay. Data are means ± SD of triplicate experiments. (C) Effect of NFATc3 knockdown on migration ability of SCC4 was determined by transwell chamber. Migration ability was described as number of migrated cells per field with data as mean ± SD for 3 randomly selected fields. Bar indicates 100 μm.
